# Ochratoxin A: 50 Years of Research

**DOI:** 10.3390/toxins8070191

**Published:** 2016-07-04

**Authors:** Frantisek Malir, Vladimir Ostry, Annie Pfohl-Leszkowicz, Jan Malir, Jakub Toman

**Affiliations:** 1 Department of Biology, Faculty of Science, University of Hradec Kralove, Hradec Kralove 50003, Czech Republic; jakub.toman.1@uhk.cz; 2 National Reference Center for Microfungi and Mycotoxins in Food Chains, Center of Health, Nutrition and Food in Brno, National Institute of Public Health in Prague, Brno 61242, Czech Republic; ostry@chpr.szu.cz; 3 Department Bioprocess & Microbial Systems, Laboratory Chemical Engineering, INP/ENSA Toulouse, University of Toulouse, UMR 5503 CNRS/INPT/UPS, Auzeville-Tolosane 31320, France; 4 Institute of State and Law, Czech Academy of Sciences, Narodni 18, Prague 11600, Czech Republic; jan.malir@ilaw.cas.cz

**Keywords:** ochratoxin A, microfungi, food, feed, toxicity, Balkan endemic nephropathy, carcinogenicity, urothelial cancer, biomarkers

## Abstract

Since ochratoxin A (OTA) was discovered, it has been ubiquitous as a natural contaminant of moldy food and feed. The multiple toxic effects of OTA are a real threat for human beings and animal health. For example, OTA can cause porcine nephropathy but can also damage poultries. Humans exposed to OTA can develop (notably by inhalation in the development of acute renal failure within 24 h) a range of chronic disorders such as upper urothelial carcinoma. OTA plays the main role in the pathogenesis of some renal diseases including Balkan endemic nephropathy, kidney tumors occurring in certain endemic regions of the Balkan Peninsula, and chronic interstitial nephropathy occurring in Northern African countries and likely in other parts of the world. OTA leads to DNA adduct formation, which is known for its genotoxicity and carcinogenicity. The present article discusses how renal carcinogenicity and nephrotoxicity cause both oxidative stress and direct genotoxicity. Careful analyses of the data show that OTA carcinogenic effects are due to combined direct and indirect mechanisms (e.g., genotoxicity, oxidative stress, epigenetic factors). Altogether this provides strong evidence that OTA carcinogenicity can also occur in humans.

## 1. Introduction

Ochratoxin A (OTA) is one of the most important and deleterious mycotoxins [1,2].

OTA was isolated and chemically characterized in 1965 [3,4]. OTA was discovered in South Africa as a toxic metabolite of *Aspergillus ochraceus* in a corn meal that was intentionally inoculated with this microfungus [3]. Further research has shown that OTA is nephrotoxic, hepatotoxic, embryotoxic, teratogenic, neurotoxic, immunotoxic, genotoxic, and carcinogenic in many species with species and sex-related differences [5,6,7,8,9,10]. The International Agency for Research on Cancer classified OTA as a possible human carcinogen (group 2B) in 1993 based on a great amount of evidence of its carcinogenity discovered in several animal studies [11]. The susceptibility to cancer is species- and sex-dependent [8,9,12,13,14,15]. Frequent exposure of animals or humans to OTA may cause a range of health problems. In particular, OTA could be a threat of cancer for humans. It will be shown further in this article that OTA acts as a nephrotoxin and an urothelial carcinogen as a result of both the oxidative stress and direct genotoxic mechanisms. Strikingly, chronic exposure to low OTA doses could be even more damaging than acute exposure to a high dose [16,17]. Humans are normally exposed to OTA—as they are to other mycotoxins—through several routes, dietary intake being the most prominent. Dermal contact or inhalation exposures are of a minor importance with respect to the general population [18], although, occasionally, these routes may also play a role [19,20].

**Figure 1:**
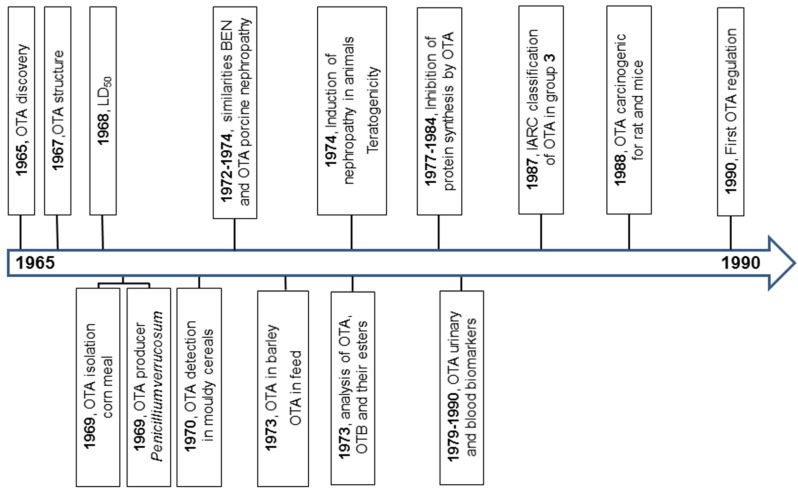
The milestones in ochratoxin A (OTA) research in years 1965–1990.

**Figure 2:**
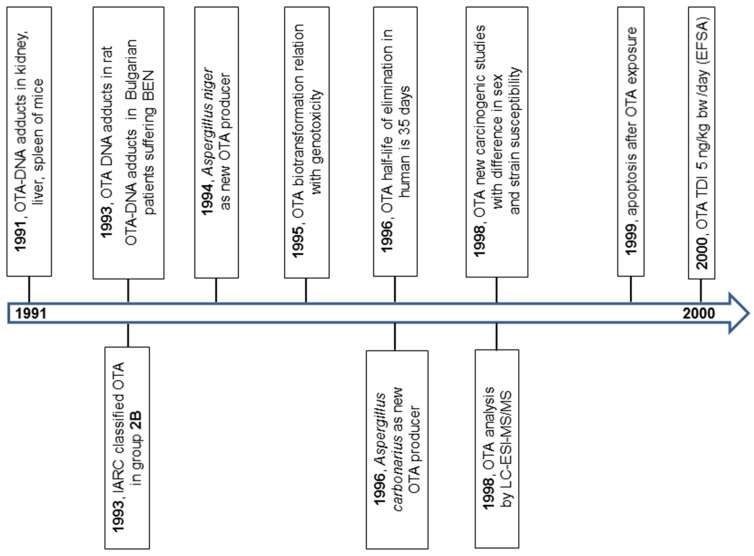
The milestones in OTA research in years 1991–2000.

**Figure 3:**
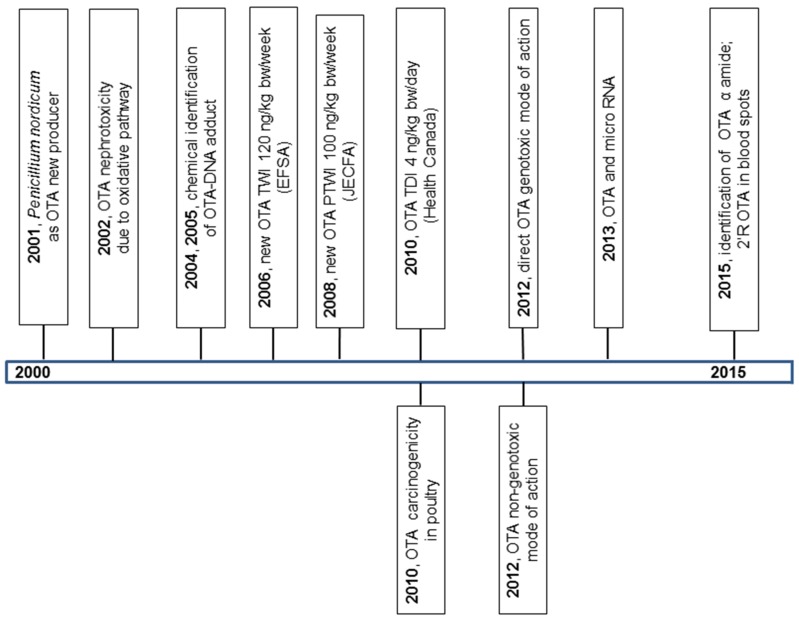
The milestones in OTA research in years 2000–2015.

In this paper, we attempt to review the data on OTA research from its discovery. The principal milestones in OTA research in 1965–1990, 1991–2000, and 2000–2015 are summarized in Figure 1, Figure 2 and Figure 3.

## 2. OTA Producers in Foodstuffs

*Aspergillus ochraceus* was the first producer of OTA ever identified. OTA was first discovered in corn meal intentionally inoculated with *Aspergillus ochraceus* [3]. Then, in a survey on OTA occurrence, producing strains isolated from feedstuffs, 2/19 isolates of *Aspergillus niger* var. *niger* were able to produce OTA in medium containing 2% yeast extract and 15% of sucrose broth, and in maize cultures. This was the first report on the production of OTA by *Aspergillus niger* [21]. Furthermore, Teren et al. (1996) tested 157 strains belonging to *Aspergillus* section *Nigri* for OTA production [22]. OTA was also detected in the culture filtrates of 5/12 *Aspergillus carbonarius* strains and 3/100 isolates in the *A. niger* aggregate (*A. foetidus* and *A. niger*). OTA-producing *Aspergillus* species, *A. carbonarius* (and the closely related *A. niger* which produces OTA more rarely), grow well at high temperatures and produce pigmented hyphae and spores, making these species resistant to UV light. Consequently, *A. carbonarius* is commonly found in grapes and similar fruits that mature in sunlight and at high temperatures [23]. The ability of *Aspergillus tubingensis* to produce OTA and the influence of grape variety on the occurrence of OTA-producing fungi in grapes were described for the first time in 2005 [24]. New OTA-producing species of *Aspergillus* section, *Circumdati* *A. westerdijkiae* and *A. steynii* isolated from coffee, were discovered in 2004 [25]. Moreover, Samson et al. (2004) found new OTA-producing species in *Aspergillus* section *Nigri—Aspergillus lacticoffeatus* and *A. sclerotioniger*—which were also isolated from coffee [26]*.*

In 1969, Walbeek et al. isolated OTA from *Penicillium viridicatum* [27]. Due to considerable revisions in taxonomy, particularly within the genus *Penicillium*, and ensuing difficulties in correct assignation, this identity has changed over the years [28]. Several authors have drawn attention to the fact that isolates of *Penicillium viridicatum* as defined at that time could be now divided into three groups depending on their various properties, including growth rates, mycotoxin production, and source [28,29]. *Penicillium viridicatum* isolates from group I grow rapidly, and they are first bright yellow green and turn forest green with age. They are mostly isolated from moldy grain but have not been found to produce either OTA or citrinin (CIT). *P. viridicatum* isolates from group II grow slowly and are yellow green both at maturity and in age. They are isolated from various plant sources, and produce both OTA and citrinin. *P. viridicatum* isolates from group III grow moderately quickly and turn brown with age. They come from meat or meatpacking plants in Europe. These latter isolates produce OTA when freshly isolated, but have not been found to produce citrinin. The taxonomy of *P. viridicatum* and *P. verrucosum* has been reviewed to clarify the conflict relating to the three *P. viridicatum* groups as laid down by Ciegler et al. (1973) [29]. It has been concluded that *P. viridicatum* group II corresponds to *P. verrucosum* and not to *P. viridicatum*, as indicated by Pitt (1979) [30]. Among species in subgenus *Penicillium*, only *P. verrucosum* is known to produce OTA. The main food habitat for *P. verrucosum* appears to be cereals growing in cool temperate zones, ranging across Northern and Central Europe and Canada [23]. In 2001, *Penicillium nordicum* was determined and confirmed as the second OTA-producing *Penicillium* species along with *P. verrucosum* [31]. Despite their shared ability to produce OTA, Larsen et al. (2001) claimed that the two species differ in several ways [31]. *P. nordicum* and *P. verrucosum* occupy different ecological niches. OTA-producing isolates originating from plant-derived material are almost always contaminated by *P. verrucosum*, whereas OTA producers in meat or cheese are derived from *P. nordicum*. Under many laboratory conditions, *P. nordicum* produces more OTA than *P. verrucosum* isolates, and lack to produce citrinin [31,32].

Table 1 and Table 2 provide an overview of the current identity of microfungi *Aspergillus* and *Penicillium* species, which are capable of producing OTA in foodstuffs [33].

## 3. OTA Chemistry

### 3.1. Chemical Characterization of OTA

CAS name (Chemical Abstracts Services) Registry No.: 303-47-9.

Chemical Abstracts: *L*-Phenylalanine, *N*-[(5-chloro-3,4-dihydro-8-hydroxy-3-methyl-1-oxo-1-*H*-2-benzopyran-7-yl)carbonyl]-,(*R*)-.

IUPAC name: (*N*-[[(3*R*)-5-chloro-8-hydroxy-3-methyl-1-oxo-7-isochromanyl] carbonyl]-3-phenyl-l-alanine).

Other name: (−)-*N*-[(5-chloro-8-hydroxy-3-methyl-1-oxo-7-isochromanyl) carbonyl]-3-phenylalanine.

Summary formula: C_20_H_18_ O_6_ClN.

**Figure 4:**
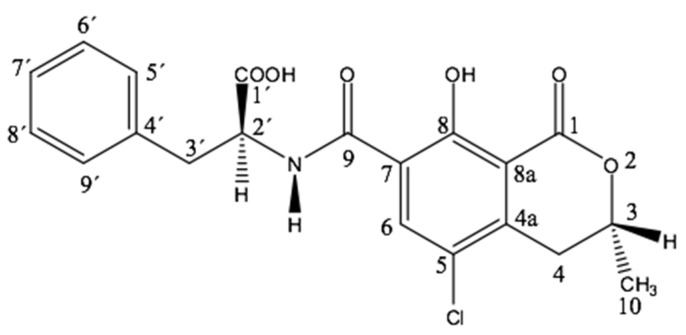
Structural formula of OTA.

OTA consists of a para-chlorophenolic moiety containing a dihydroiso-coumarin group that is amide-linked to L–phenylalanine. See Figure 4 and Table 3 for structure of the OTA derivatives.

Molecular Weight: 403.8.

Chemical and physical properties of OTA were comprehensively described by Budavari (1989) [34] and IARC (1993) [11], its melting point was determined by van der Merwe et al. (1965) [3,4] and Kuiper-Goodman and Scott (1989) [35], and its optical rotation by Pohland et al. (1982) [36]. Spectroscopic data on OTA (as ultraviolet, infrared, mass spectral and proton nuclear magnetic resonance data) were reported by van der Merwe et al. (1965) [3,4] and Pohland et al. (1982) [36], OTA solubility (e.g., in chloroform, ethanol, methanol, xylene) by WHO (1990) [37], and its stability (partial degradation under normal cooking conditions) by Müller (1982) [38]. OTA degradation was performed by treatment with an excess of sodium hypochlorite solution [39]. Physico-chemical properties of OTA and the progress in their knowledge have been recently reviewed in great detail by Khoury and Atoui (2010) [40]. OTA is a weak acid with two pka (4 and 7) [41].

Table 3 described several derivatives occurring naturally or formed in the body after biotransformation. Some are hydroxylated, others lack phenylalanine moiety or are conjugated (e.g., with glutathione, glucuronic acid, sulfate, or pentose) [40,42,43,44,45,46,47,48,49,50].

The most recently discovered ones include a decarboxylated hydroquinone derivative, DC-OTHQ (often linked to glutathione) [43,63,64,65].

During coffee roasting (at 225 °C), 2′-DC-OTA and 2′*R*-OTA, two products of thermal degradation of OTA, were identified [66]. Ochratoxin α amide, which is formed at high temperatures during coffee roasting, was discovered. This represents another product of thermal degradation of OTA [67].

## 4. OTA Analysis

Principal methods developed for OTA determination in biological materials are summarized in Table 4.

In fact, more sensitive analytical methods or new methods for determining OTA and ochratoxins in biological materials are being developed consecutively toward the sophisticated development of instrumentation and analytical techniques but also toward the improvement of laboratory analytical methods. The most used and traditional analytical techniques include thin-layer chromatography, HPLC, and ELISA. Therefore, in the present article, the analytical techniques are divided into traditional ones, and the others. 

Generally, all chemical methods for the analysis of OTA consist of several steps (extraction, clean-up, separation, detection, quantification, and confirmation of identity) [100]. Conventional sample extraction and clean-up are usually achieved by liquid extraction for OTA determination in kidneys of swine [101]. More recently, solid-phase extractions (SPE) notably for OTA determination in animal feed [102] and immunoaffinity columns (IAC) [103,104] (/homemade of IAC/; immunoaffinity cartridges commercially available) have become popular [105]. At present, different kinds of cartridges are commercially available for clean-up and pre-concentration, including IAC and molecular imprinted polymers (MIPs) cartridges, composed by anti-OTA antibodies and three-dimensional network specific for the target molecule. In this case, OTA passed through cartridges (e.g., Mycosep™ or Mycospin™) [106]. It is based on adsorption and the ion-exchange process [107]. The use of immunoaffinity chromatography in the clean-up step improves mycotoxin analysis and has a number of advantages: clean extracts, precision and accuracy, rapidity, and reduction of the use of dangerous solvents [82]. The main advantages of these columns are the specific binding of OTA onto the antibody and the near-complete removal of matrix interference [108]. Nevertheless, in the case of OTA, underestimation can be observed if extraction is done in an alkaline condition, because OTA is converted into open-ring OTA (OP-OA) and no longer recognized by antibodies [109,110,111,112].

The confirmation of OTA presence in biological materials is very important in order to guarantee quality of analytical results. Hult and Gatenbeck (1976) presented the OTA confirmation with carboxypeptidase A [70], as did Hunt et al. (1980) with boron trifuoride methanol [73] and Studer-Rohr et al. (1995) with diazomethane [113]. Quality assurance of analytical results (a laboratory accreditation, participation in proficiency testing, and the use of certified reference materials) according to the past norm EN 45001 (1989) [114] and the recent norm which is in force EN ISO/IEC 17025 (2005) [115] is very important for the purposes of OTA determination in biological materials.

Many analytical methods for the determination of OTA have been developed over time [100], and most of them involve the use of thin-layer chromatography (TLC) [68,69] and, predominantly, high-performance liquid chromatography (HPLC) with fluorescence detection (FLD) [72]. Subsequently, OTA is identified and detected by LC-MS [77], LC-MS/MS [83,84]*,* aptamers [88,92,116], ELISA [76,117], and immunosensing methods [118]. However, the technique most commonly used is based on liquid chromatography (LC) coupled with a fluorometric detector for highly sensitive detection signal [106]. It is known that, due to natural OTA fluorescence, OTA is generally determined by chromatographic techniques [119,120]. 

The other methods for the OTA determination used include gas chromatography–mass spectrometry (GC-MS) [79,113], fluorometric kits (the immunoaffinity columns coupled with a fluorometer) [82,87], fluorescence polarization immunoassay (PFIA) [87], isotope dilution [121], and a radioimmunoassay (RIA) [75,122,123,124,125,126]; however, due to health hazards of radiolabeled compounds and specialized waste disposal, RIA has not been in use for a long time [127]. More recent methods for OTA determination are inductively coupled plasma mass spectrometry ICP-MS [90], and capillary electrophoresis techniques [128]: capillary electrophoresis with laser-induced fluorescence detection (CE-LIF) [129,130], micellar electrokinetic capillary chromatography/MEKC/ [131], molecular imprint polymers (MIPs) [132,133,134], biosensors [133,135,136], and aptamers (single-stranded oligonucleotides (DNA or RNA) selected in vitro to bind with high affinity and specificity to molecular targets) [88,92]. The applications of aptamers are known and developed, e.g., in chromatography, capillary electrophoresis, mass spectrometry, and biosensors [137,138].

## 5. Occurrence of OTA in Food and Feed

In 1969, Shotwell et al. [139] with colleagues from the U.S. Department of Agriculture (USDA) published the very first piece of information about the amount of OTA in a maize sample at levels from 110 to 150 ng/g. In 1970, Scott et al. [140] from Health Canada published data on OTA in moldy cereals, beans, and peanuts. OTA concentrations in wheat, oats, barley, and rye (62.0% positive samples) ranged from 30 to 27.000 ng/g [140,141]. The occurrence of OTA in pig kidney was first mentioned by Hald and Krogh in 1972 [142] and by Hunt et al. in 1979 [143]. Since that time, more than 90 kinds of foodstuffs of both plant and animal origins, including milk, have been found to contribute to the OTA dietary exposure [33].

As for foodstuffs of plant origin, OTA occurs in cereal products, olives, beans, beer, wine, coffee, cocoa products, raisins, figs, licorice, pulses, pumpkin seeds, and tea. In general, the average concentration of OTA is reported to range from 0.1 to 100 ng/g. OTA concentration in black pepper, cayenne pepper, caraway, cardamom, coriander, chili powder, curcuma, and dried red pepper ranges from 1 to 100 ng/g. Feedstuffs of plant origin—those made of wheat, oats, barley, rye, maize, rice, millet, sorghum, soybean, horse bean, peas, bean, broad bean, alfalfa, sunflower or pumpkin seeds, coconut, peanut cake, and hay/silage—also contain from 1 to 100 ng/g of OTA [144,145,146].

In foodstuffs of animal origin, e.g., in pork blood products, edible offal, pork meat, chicken meat and offal, and dry-cured ham, the levels of OTA range from 0.1 to 1 ng/g. The same amounts are measured in feedstuffs of animal origin, e.g., in pork kidney and liver, pork meat, chicken liver, and viscera, and in mechanically separated chicken used as ingredients in pet food for cats and dogs [144,145,147].

Table 5, Table 6 and Table 7 summarize the recent data related to OTA in foodstuffs obtained from the EU Rapid Alert System for Food and Feed (RASFF) [146]. The RASFF dealt with OTA in 175 cases in 2000–2015.

## 6. OTA Toxicity

### 6.1. OTA Nephrotoxicity

OTA has been found to cause porcine and poultry nephropathy. OTA is implicated in the pathogenesis of some renal diseases including Balkan endemic nephropathy (BEN), kidney tumors occurring in certain endemic regions of the Balkan Peninsula [14,148], and chronic interstitial nephropathy (CIN) occurring in Tunisia [149,150] and other North African countries [151].

Kidney lesions have been observed on proximal tubules. The epithelial cells were damaged, for example, membrane integrity was lost, and the size and the density of the brush border were reduced. The chromatin was condensed, and the nuclear envelope disappeared. The histologic picture shows an enlargement of tubular membrane and an apparition of collagen fibers [152].

At the beginning, the BEN disease is characterized by a modification to epithelial cells without any change in the size of the organ. After chronic exposure, kidneys are reduced and interstitial fibrosis is the most important picture. At the end stage, impairment of renal function leads to enzymuria (e.g., gamma glutamyl transferase, alkaline phosphatase, lactate dehydrogenase) [153], polyuria accompanied with red tongue, thirst, and bitter taste [153]. Neither edema nor hypertension can be observed. Other symptoms such as headaches, lumbar pain, asthenia, and anemia (iron deficiency) were recorded. Several biochemical parameters changed including glycosuria, proteinuria (0.15–0.5 g/ 24 h), alkalinization of urine, elevated serum creatinine, and an increase in immunoglobulin M (IgM) and immunoglobulin E (IgE) [154,155].

Data on OTA nephrotoxicity are summarized in Table 8.

### 6.2. OTA Carcinogenicity 

Data on OTA carcinogenicity are summarized in Table 9.

In 1976 and 1983, IARC first evaluated the carcinogenic risk that OTA poses toHuman. No report on cases of cancer or epidemiological studies were available at that time and, in the absence of adequate epidemiological data, no evaluation of the carcinogenicity of OTA with respect to Humans could have been made [225,226]. In 1987, the IARC reclassified OTA into Group 3 (not classifiable for its carcinogenicity to humans). Based on a great amount of evidence of OTA carcinogenicity revealed in new animal studies, it was again reclassified into Group 2B (possibly carcinogenic to humans) in 1993. At present, new information regarding genotoxicity of OTA (formation of OTA-DNA adducts), its role in oxidative stress, and the identification of epigenetic factors involved in OTA carcinogenesis—should they indeed provide strong evidence that OTA carcinogenicity is mediated by a mechanism that also occurs in humans—could lead to another reclassification of OTA. In the light of recently available data, it does not seem inappropriate to upgrade its carcinogenicity from Group 2B (possibly carcinogenic to humans) to at least Group 2A (probably carcinogenic to humans) [227] or, in our opinion, even to Group 1 (carcinogenic to humans).

## 7. OTA Biomarkers

Biomonitoring of OTA provides the best approach to assess the human exposure to OTA from any source and through any route [228]. The first studies reporting the presence of OTA in human blood were carried out in the Balkans in the 1970s [229]. The exposure of the human population to OTA and other ochratoxins represents a worldwide problem. Baldwin et al. (2011) reviewed biomarker researches for the most important mycotoxins and defined biomarkers [230]. Recently, a biomarker of exposure has been defined to be a biological measure which is correlated with the quantity of the xenobiotic ingested; resulting in the improved exposure classification in comparison with more traditional approaches [231]. OTA in milk (non-invasive sampling), OTA in blood serum (invasive sampling), OTA in urine (non-invasive sampling), and OTA in human kidneys (sampling post-mortem or after nephroctomia) are qualified as biomarker of exposure to OTA [232]. Soto et al. (2015) have recently used several biomarkers for evaluating the OTA exposure. The values of OTA detected in potential biomarkers of exposure for blood, breast milk, and urine ranged from 0.15 to 18.0, from 0.002 to 13.1, and from 0.013 to 0.2 ng/mL, respectively. The calculated EDI for OTA in plasma ranged from 0.15 to 26 ng/kg bw/day and has turned out to be higher than that obtained in urine (0.017 to 0.4 ng/kg bw/day). All these values have been correlated with the range of EDI for OTA calculated from food products: 0.0001–25.2 ng/kg bw/day [233].

### 7.1. OTA in Human Blood 

In 1979, OTA determination in human whole blood and serum was developed [234]. In the past several decades, OTA has been detected in human blood samples on a worldwide scale. Scott (2005) has described OTA in blood serum as a uniquely useful biomarker of OTA exposure due to its high-affinity binding to serum albumin or to other small proteins, which should result in higher serum OTA levels and long persistence of OTA in blood serum [235]. OTA blood amounts will integrate exposure over longer periods [236]. The use of serum or plasma has been described as more suitable matrices in comparison to whole blood [105,237]. Generally, the determination of OTA in blood samples remains the basic method of how to monitor human exposure to OTA, which is ubiquitous in human blood serum/plasma and indicates continuous exposure to the toxin, originating mainly from food intake [235]. 

Table 10 describes some of the most notable findings of OTA in blood on a worldwide scale.

Advantages arising from monitoring OTA in the blood of healthy persons consist mainly in relatively high OTA levels found compared with OTA determinations in urine [232]. OTA blood determination will integrate exposure over longer periods, while biomarker analysis in urine apparently better reflects day–to-day variations in the exposure of adults and infants [231,236,237,238,239,240].

### 7.2. OTA in Urine

Urine is a major excretion route for both OTA and OTα (5-chloro-8-hydroxy-3-methyl-1-oxo-3,4-dihydroisochromene-7-carboxylic acid; formula, see Table 3) in humans [45]. OTA can be found in urine several days after OTA ingestion [8]. The elimination of OTA through human urine has been reported to be low (mean value between 20 and 80 ng/day) and independent of the dose ingested [237]. The OTA uptake has been described as dependent on the free OTA concentration, which is severely limited by the binding of OTA to serum albumin [8]. Thus, the relationship between OTA in urine and OTA intake remains a complex issue as in the case of OTA in blood.

The first study measuringing OTA in urine in Europe was conducted by Mac Donald et al. (2001) [270] in the UK. In this study, OTA was found in 46 urine samples (92%) collected over 24 h from 50 volunteers (healthy individuals from the UK). OTA concentrations ranged from <10 to 58 ng/L, and the mean value was about 21 ng/L. This study demonstrated a strong correlation between OTA concentrations in urine and its dietary intake. The second study in Europe was conducted in Bulgaria by Castegnaro et al. (1991) [303]. A total of 152 urine samples collected from patients with BEN (Balkan endemic nephropathy) or urinary tract tumors (UTT) and from the control families were analyzed, and OTA was detected in about 33% of the samples of urine (more often in endemic villages than in nonendemic ones) in the range 5–604 ng/L and in healthy people in the range 5–43 ng/L (LOQ = 5 ng/L). In Europe, another one-month follow-up study of OTA in urine samples after a 24-h collection of urine from the inhabitants with BEN in Bulgaria (from 16 healthy volunteers from two villages located in the Vratza district with a high risk area for BEN; 5 of Gorno Pestehne, 11 of Beli Izvor) was conducted by Petkova-Bocharova et al. (2003) [85]. 98% of samples were positive and contained OTA in the range 10–1910 ng/L. The OTA mean value in Gorno Pestehne was 50.8 ng/L, and in Beli Izvor it was 168.6 ng/L [85]. In a Czech study carried out in 2010, OTA was measured in a total of 236 samples of urine collected from healthy persons within a 24-h cycle (males/females, 45–60 years old, two samples per person from non-consecutive days, with at least a 14-day time difference). A total of 185 samples (78%) of these 236 samples were positive, with a limit of quantification (LOQ) of 2.0 ng/L, a mean of 7.32 ng/L, and a median of 4.47 ng/L [304,305]. These data signalize the real exposure of the given population group to OTA, with a higher percentage of positive urine samples in men (92%) than women (65%) [305]. 

OTA was usually determined in morning urine (not 24-h urine) in these countries (see also Table 11). However, in exposure studies, it is recommended that urine is collected over 24 h—representative of the excretion throughout a day [306].

The multibiomarker methods have been applied in several pilot studies to prove their applicability and to estimate mycotoxin exposure in the populations/individuals tested. The application of these methods resulted in advanced data on exposure patterns and revealed new findings on co-exposure to the mycotoxin combinations [307]. In addition, it must be mentioned that urinary excretion mainly reflects the recent mycotoxin intake, whereas measurements in plasma/serum are more likely to reflect the long-term exposure [307]. As a result of the advent of the latest generation of high-performance LC-MS/MS instruments, a clear trend toward the development and application of multianalyte methods in mycotoxin biomarker research can be observed [308]. Warth et al. (2012) injected samples directly into the LC-MS/MS system to facilitate the quantification of 15 analytes [308]. A method developed by Ediage et al. (2012) [91] covered seven mycotoxins and several important conjugation and breakdown products (for a total of 18 analytes). In this study, OTA, OTα, and 4-OH OTA were measured in human volunteers [91]. However, none of the target metabolites of OTA such as OTα or 4-OH OTA were confirmed in another study performed with urine samples in Cameroon [309], but the data correlate with similar findings reported for a Korean population [89]. According to Munoz et al. (2010a) [276], interindividual variability in the detoxification of OTA in human urine may account for the observed variations in urinary OTα, and the possibility cannot be excluded that a low rate of OTA detoxification is a characteristic of some human populations [309]. The highest concentration of OTA reported so far in human urine was detected in Sierra Leone with a range of 70–148,000 ng/L, but no mean was reported [310]. Table 11 summarizes the OTA detection in human morning urine around the world. Last but not least, in dietary studies carried out in Serbia, in addition to OTA, several OTA derivatives have been detected in urine (and in blood). A clear difference between men and women has been observed [17].

### 7.3. OTA in Human Milk 

As OTA is also excreted via human milk, breastfed children including babies are exposed to OTA as well [239,330]. Nevertheless, OTA amounts in milk are reported to be much lower than concentrations of OTA in blood (down to 10 times) [331]. In Italy, OTA was detected in milk from healthy women with varying daily diets in different geographical regions [332]. The relationship between OTA contamination of human milk and its dietary intake was examined [333], and it was confirmed that OTA occurrence in human milk was likely associated with maternal dietary habits. The strongest associations were observed with foodstuffs of plant origin and, to a lesser extent, with foodstuffs of animal origin [333].

Table 12 summarizes data on OTA presence in human milk worldwide.

In some countries, e.g., Egypt, Turkey, and Sierra Leone, OTA milk concentrations were found to be more than 100-fold higher in comparison with Europe (see Table 12). It can be concluded that, despite the fact OTA concentrations in milk compared with blood are much lower, OTA contamination of human breast milk presents a potentially serious health hazard [354].

### 7.4. OTA in Human Kidneys 

OTA presence in human tissues seems to be direct and definite proof of human exposure to OTA, although practicability of such measurements “in vivo” is obviously limited [355]. Taking OTA’s nephrotoxicity in mind, in particular, there are not many studies available that have attempted to determine OTA in human kidneys. Several studies have been carried on the content of OTA in human kidneys, e.g., in Germany [356], in the Czech Republic in 30 samples of kidney (40% positive/detectable/samples; OTA ranged from 0.1 to 0.2 ng/g; mean 0.07 ng/g; results of OTA < 0.1 ng/g (LOQ) given as 1/2 limit of quantification = 0.05 ng/g) [357], and in Poland in 19 samples of kidney (78.9% positive/detectable; OTA ranged from 0.15 to 0.39 ng/g with mean 0.26 ng/g) [268]. Several human kidneys samples (60) obtained from patients suffering from kidney (or urinary bladder) cancer from Bulgaria (8 samples) [186], Serbia (10 samples), Croatia (16 samples), and France (18) [16,17] have been analyzed up to now. Not only was OTA detected but also OTA derivatives such as OTHQ, OTHQ-GSH, 4-OH OTA, and OTB. Interestingly, DNA adducts were detected, and the nature of the DNA was in relation to the OTA derivatives. In Croatia, the DNA adducts profile of a farmer was similar to the profile of the pigs and poultry from his farm. It has been observed that the exposure has been higher in rural regions, and co-exposure to CIT and/or FB has been systematic [16,17].

## 8. Regulation of OTA in Food and Feed

Due to its toxic properties, OTA is subject to legal regulation both on national and international levels. The toxicity of OTA became more or less evident by the end of the 1970s. A real debate on whether OTA in food and feed shall be regulated on a national or international level does not seem to predate the 1990s. This circumstance contrasts with the case of other mycotoxins, in particular, the aflatoxins (in the USA, the first limits for aflatoxins were established as early as the 1960s; soon after their discovery [358], the European Communities followed in the 1970s) [359].

**Figure 5:**
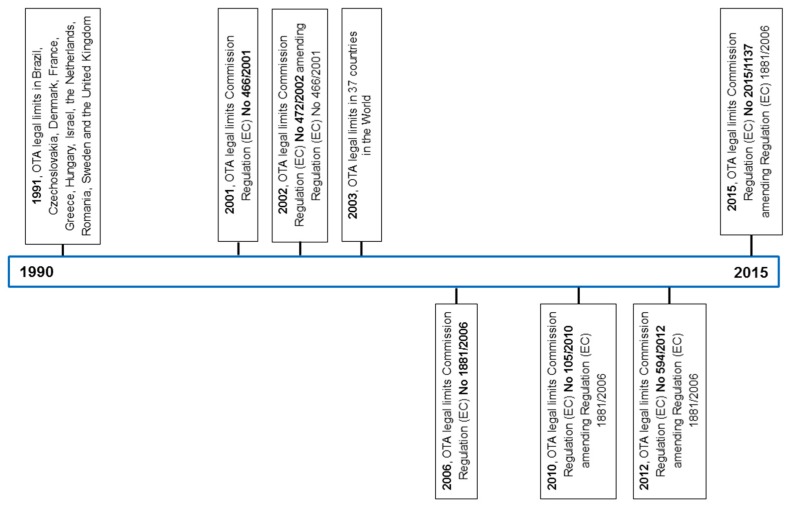
The milestones in evolution of legal regulation of OTA in years 1965–2015.

For OTA, in 1991, van Egmond estimated that in 60 countries where some legal regulations with respect to mycotoxins existed, only 11 set limits on OTA (Brazil, Czechoslovakia, Denmark, France, Greece, Hungary, Israel, The Netherlands, Romania, Sweden, and the United Kingdom) [360]. In 2003, when a worldwide survey on legal regulation of mycotoxins was conducted by the FAO in cooperation with the Dutch Foreign Service, the number of countries with legal limits on OTA in food and feed grew to 37 (compared to more than 76 countries with legal limits for aflatoxins) [359]. No such large-scale survey has been reported ever since [361]. However, it may be assumed that the number of countries where OTA presence in food and feed is subject to legal regulation is not lower now than it was in 2003 (see Figure 5). This assumption can be based on two major arguments. Firstly, since 2003, research has provided new data on OTA’s harmful effects to human and animal health. Secondly, due to the globalization of food and feed markets, discussion on how to tackle the health hazards linked to OTA (and other mycotoxins) has intensified on an international level and has had repercussions back on the national level. By way of example, China seems to have recently established limits on OTA in both food and feed [362].

Membership of States in international or regional organizations may also contribute to adoption of legal regulations on OTA. For the time being, the binding maximum limits on OTA appear to exist only in the European Union (EU) (see infra). On the global level, debate on the feasibility of establishing the maximum limits on OTA has taken place at the Codex Alimentarius Commission (CAC), the joint intergovernmental body established by the FAO and WHO responsible for implementing the Joint FAO/WHO Food Standards Programme. After the Joint Food and Agricultural Organization (FAO)/World Health Organization (WHO) Expert Committee on Food Additives (JECFA), an expert body which provides scientific advice to the CAC repeatedly dealt with OTA in 1991, 1995, 2001, and 2007, the maximum limit of 5 µg/kg with respect to wheat, barley, and rye has been recently established under the Codex General Standard for Contaminants and Toxins in Food and Feed [363]. In addition, there are four codes of practice that aim at the prevention and reduction of OTA contamination in cereals [364], wine [365], coffee [366], and [367] adopted between 2007 and 2014 [368]. Although the Codex Alimentarius standards are not per se binding, their importance stems especially from the fact the World Trade Organization (WTO) considers the measures taken by its Member States in conformity with the Codex Alimentarius standards to be science-based, appropriate, and nondiscriminatory under the WTO Agreement on the Application of Sanitary and Phytosanitary Measures signed in 1994 and thus does not treat them as breaches of world trade rules. 

As far as the existing limits on OTA are concerned, those of the EU are generally assessed to be the most comprehensive and detailed [359].

As for the limits on OTA in food, these were first established on the EU level by the Commission Regulation (EC) No 472/2002 [369] of 12 March 2002 amending Regulation (EC) No 466/2001 [370] setting maximum levels for certain contaminants in foodstuffs (see Table 13). As the Regulation No 466/2001 was repeatedly amended, in 2006, it was replaced by completely a new act, Commission Regulation (EC) No 1881/2006 of 19 December 2006, setting maximum levels for certain contaminants in foodstuffs [371]. The adoption of Regulation No 1881/2006 was based on the scientific opinion of the Scientific Panel on contaminants in the Food Chain of the EFSA adopted on 4 April 2006, which updated the earlier opinion of the Scientific Committee on Food on OTA adopted on 17 September 1998 [372].

In the EU, the Regulation 1881/2006 remains in force today, although it has been amended nearly 26 times. As of February 2016, the Regulation No 1881/2006 sets the maximum limits on OTA not only in cereals (both in the unprocessed cereals and cereal products) but in a wide variety of other food commodities as well (see Table 14). These limits are legally binding on all 28 EU Member States, which are obliged to apply these rules in full.

Apart from setting binding limits on OTA in food, since 2002, the EU has also unified the methods of sampling and analysis for purposes of the official control of the levels of mycotoxins in foodstuffs performed by the authorities of the Member States (first by the Commission Directive 2002/26/EC of 13 March 2002, later replaced by the Commission Regulation (EC) No 401/2006 of 23 February 2006 which remains in force today).

As for OTA in feed, however, up to now, only a non-binding recommendation exists with respect to cereal feed, and feed for pigs and poultry on the EU level (Commission Recommendation 2006/576/EC [373] of 17 August 2006 on the presence of deoxynivalenol, zearalenone, OTA, T-2 and HT-2, and fumonisins in products intended for animal feeding). For details, see Table 15.

There are, however, approaches to legal regulation of OTA other than establishing and enforcing the binding maximum limits on OTA in food and feed commodities as in the EU. Most notably, no binding limits on OTA in food or feed exist in the USA. Even more strikingly, no advisory or regulatory action limits have been established by the US authorities. Instead, the US Food and Drug Administration (FDA), acting under the Federal Food, Drug and Cosmetic Act (FFDCA), has instead consistently relied on laying down good agricultural and manufacturing practices and on requiring the implementation of food safety plans in food industry undertakings [358]. In extension, the FDA monitors the compliance with these practices and the presence of OTA in domestic and imported foods (Food Compliance Programme No 7307.001 entitled “Mycotoxins in Domestic and Imported Foods”). An approach analogous to that of the USA has been adopted by a range of other countries such as Australia, Canada, and Japan [374].

For some authors, the US approach to regulating mycotoxins including OTA is clearly preferable because it is seen as an option that might “diffuse trade frictions, and at the same time help reduce economic losses from mycotoxin contamination and divergent standards” [375]. The truth is that the US approach seems to exert a non-negligible influence on the international level, e.g., within the CAC, which has, as mentioned above, adopted four codes of good practice with the aim of reducing the OTA occurrence in several food commodities that are commercially important. 

To sum up, 50 years after the discovery of OTA, differences in how to legally regulate mycotoxins including OTA are still marked. However, even in an era when further liberalization of world trade is envisaged (e.g., a project of the Transatlantic Trade and Investment Partnership and the TTIP between the USA and the EU), due to economic and political controversies linked to the existing policies on mycotoxins, it cannot be expected that some harmonized approach to legally regulating mycotoxins including OTA will be easily established on a global level [375,376,377].

## 9. Conclusions

OTA is ubiquitously found all over the world in many foodstuffs and feedstuffs. OTA is recognized for its nephrotoxicity and, to date has been identified as one of the most potent renal carcinogens in rodents ever studied by the National Cancer Institute/National Toxicological Program (NCI/NTP) [181]. OTA is deleterious for the pig and poultry industries. For human beings, many authors consider it to be the main contributor in the pathogenesis of Balkan endemic nephropathy and some nephropathies in other parts of the world.

The development of effective strategies alleviating OTA-induced toxicity is very complex because the mechanism of action of OTA is still unclear. The toxic effect is the result of many effects such as the inhibition of protein synthesis, direct genotoxicity, and cell cycle arrest. Inhibition of OTA uptake and stimulation of OTA elimination of the body preventing OTA accumulation will be promising approaches [378].

**Figure 6:**
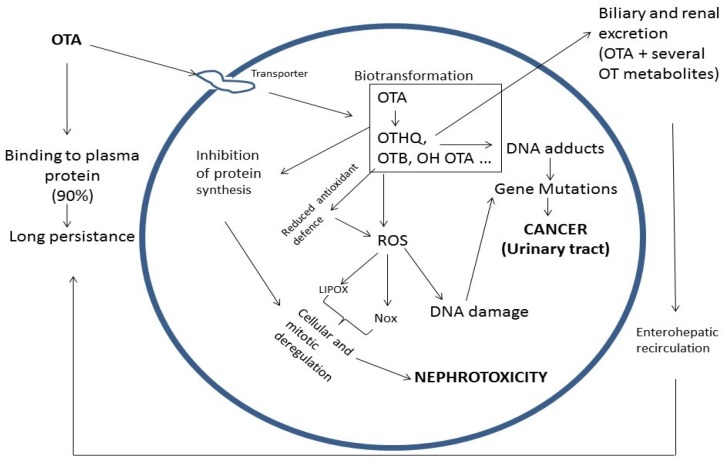
Summary of biochemical effects of OTA. Explanations: OTA: Ochratoxin A; OTHQ: Hydroxyl quinone ochratoxin; OTB: Dechlorinated ochratoxin; LIPOX: Lipoperoxidation; Nox: Nitrogen oxide; ROS: Reactive oxygen species.

**Figure 7:**
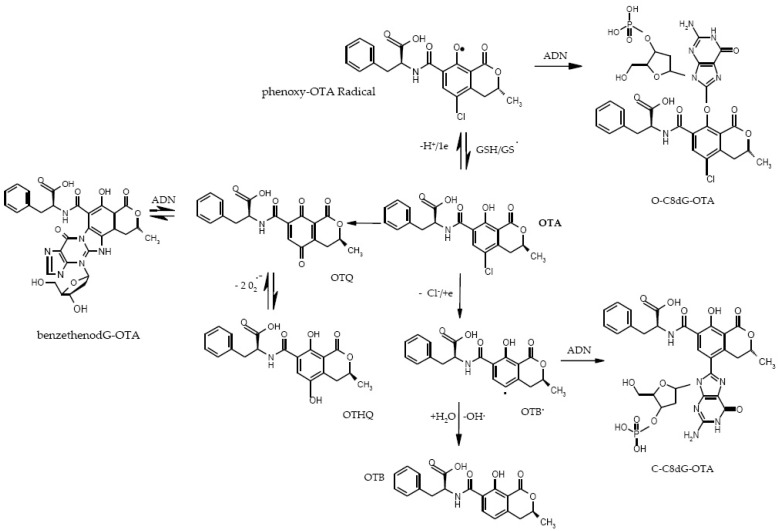
Metabolic activation of ochratoxin leading to DNA adducts. OTA: Ochratoxin A; OTHQ: Hydroxyl quinone ochratoxin; OTQ: Quinone ochratoxin; OTB: Dechlorinated ochratoxin; GSH: Reduced glutathione; GS: Oxidized glutathione; dG-OTA: Guanine OTA adduct.

Since its discovery in 1965, numerous studies have been performed with respect to OTA, which have permitted the establishment of different mechanisms for OTA nephrotoxicity and carcinogenicity (summarized in Figure 6 and Figure 7). The mechanisms leading to OTA nephrotoxicity, its hepatotoxicity and immunotoxicity, can be linked to inhibition of protein synthesis, lipoperoxidation, and modulation of MAP kinase cascade (Figure 6), whereas its carcinogenicity arises after the metabolic activation of OTA in a way similar to pentachlorophenol derivatives (Figure 7).

OTA forms covalent DNA adducts through radical and benzoquinone intermediates. The OTHQ metabolite of OTA can undergo an autoxidative process to generate the quinone electrophile OTQ that reacts with DNA. In addition, the formation of OTQ or phenoxy and aryl radicals can lead to increased ROS production that causes cytotoxicity. Radical species generate a C-bound C8-dG adduct, while benzetheno-type DNA adducts are expected from the quinone electrophile. The quinone-type adducts form faster in cells and stem from P450 activation of OTA. The C-bound C8-OTA adduct forms at a slower rate and is predicted to stem from reductive dehalogenation of OTA (via GSH and cyclooxygenase or lipoxygenase). The C5-Cl atom is critical for DNA adduction (genotoxicity) but not for cytotoxicity (OTB is cytotoxic but not genotoxic) (Figure 7).

Several quinone derivatives have been isolated from blood and urine and also in human or animal tissues exposed to OTA. The OTB-dG adduct is consistently found by ^32^P-postlabeling in kidney DNA from OTA-treated rats, pigs, and humans. These metabolites and this adduct could serve as biomarker for OTA exposure.

Increases in carcinogenicity and genotoxicity during co-exposure with citrinin (CIT), fumonisin (FB), or both can be explained by both factors. FB and CIT induce COX2, thus favoring the biotransformation of OTA into a genotoxic compound. Moreover, the quinone methide structure of CIT could easily explain the generation of DNA adduct. It may be capable of oxidizing OTA into the phenoxyl radical to promote C-C8 adduct formation. The new findings on OTA mutagenicity favor direct genotoxicity and rule out oxidative DNA damage as a contributor to the induction of deletion mutations or renal carcinogenesis. Therefore, further research should focus on co-exposure.

Altogether, OTA is a complete carcinogen, active since the earliest stage of life. Intake evaluation based on real analysis shows that the daily intake was three times greater than the virtual safety dose of 4 ng/kg bw/day—against carcinogenicity (intake per day 648 ng/60 kg adult) [379].

Maternal-fetal risk assessment of OTA during pregnancy was conducted using the benchmark dose approach for genotoxic carcinogens. Considering the sensitivity of a fetus, risk reduction is a high priority. It is essential to keep exposure to OTA as low as possible in women, notably during pregnancy [380].

Among the professional community, it is agreed that OTA is one of the five most agriculturally important mycotoxins; therefore, continued attention must be paid to research on ochratoxins and OTA in order to elucidate their metabolism, genotoxicity, and mechanism of action for renal carcinogenicity, with the ultimate aim of protecting public health and preventing economic losses.

## Figures and Tables

**Table 1:** *Aspergillus* species as OTA producers in foodstuffs.

Genera	Section	Species	Foodstuffs (Examples)	Year of Discovery
*Aspergillus*	*Circumdati*	*A. ochraceus* G. Wilh.	Soya bean, nuts, red pepper, cereals, green coffee beans	1965
		*A. steynii* Frisvad & Samson	Coffee beans	2004
		*A. westerdijkiae* Frisvad & Samson	Coffee beans	2004
	*Nigri*	*A. carbonarius* (Bainier) Thom	Grapes, red pepper, coffee beans	1996
		*A. foetidus* Thom & Raper	Grapes	1996
		*A. lacticoffeatus* Frisvad & Samson	Coffee beans	2004
		*A. niger* Tiegh.	Grapes, peanuts	1994
		*A. sclerotioniger* Frisvad & Samson	Coffee beans	2004
		*A. tubingensis* Mosseray	Grapes	2005

**Table 2:** *Penicillium* species as OTA producers in foodstuffs.

Genera	Subgenus	Series	Species	Foodstuffs (Examples)	Year of Discovery
*Penicillium*	*Penicillium*	*Verrucosa*	*P. verrucosum* Dierckx	Cereals	1969
		*Verrucosa*	*P. nordicum* Dragoni & Marino	Dry ham, salami	2001

**Table 3:**
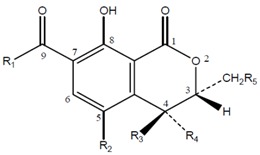
Chemical structures of OTA and its derivatives.

Metabolites	Abbreviations	MW	R1	R2	R3	R4	R5	R6	References
Ochratoxin A	**OTA**	403	Phe	Cl	H	H	H	OH	[3,4]
Ochratoxin B	**OTB**	370	Phe	H	H	H	H	OH	[51]
Ochratoxin C	**OTC**	431	Phe Ethyl ester	Cl	H	H	H	OH	[52]
Ochratoxin α	**OT**α	256	OH	Cl	H	H	H	OH	[53]
Ochratoxin β	**OT**β	223	OH	H	H	H	H	OH	[54]
4*R*-hydroxy Ochratoxin A	**4*R*-OHOA**	419	Phe	Cl	H	OH	H	OH	[55]
4*S*-hydroxy Ochratoxin A	**4*S*-OHOA**	419	Phe	Cl	OH	H	H	OH	[55]
10-hydroxy Ochratoxin A	**10-OHOA**	419	Phe	Cl	H	H	OH	OH	[56]
Ochratoxin A open lactone	**OP-OA**	421	Phe	Cl	H	H	-	OH	[57]
Ochratoxin B open lactone	**OP-OB**	388	Phe	H	H	H	-	OH	[57]
Ochratoxin α open lactone	**OP-OT**α	274	OH	Cl	H	H	-	OH	[57]
Ochratoxin β open lactone	**OP-OT**β	241	OH	H	H	H	-	OH	[57]
Ochratoxin A quinone	**OTQ**	383	Phe	O	H	H	H	O	[58]
Ochratoxin A hydroquinone	**OTHQ**	385	Phe	OH	H	H	H	OH	[58]
OTHQ decarboxylated	**DC-OTHQ**	366	Decarboxylated Phe	OH	H	H	H	OH	[43]
Conjugate Ochratoxin A quinone–glutathion	**OTQ-Glutathion**	689	Phe	O	H	H	H	O	[59]
Conjugate Ochratoxin A–acyl hexose	**Acyl-hexose-OTA**	565	Phe acyl hexose	Cl	H	H	H	OH	[60]
Conjugate Ochratoxin A–acyl pentose	**Acyl-pentose OTA**	535	Phe acyl pentose	Cl	H	H	H	OH	[60]
Ochratoxin A methyl ester	**OTA-Me**	417	Phe methyl ester	Cl	H	H	H	OH	[57]
Ochratoxin B methyl ester	**OTB-Me**	384	Phe methyl ester	H	H	H	H	OH	[57]
Ochratoxin B ethyl ester	**OTB-Et**	398	Phe ethyl ester	H	H	H	H	OH	[57]
4*R*-hydroxy Ochratoxin A methyl ester	**4*R*-OHOA-Me**	433	Phe methyl ester	Cl	H	OH	H	OH	[57]
10-hydroxy Ochratoxin A methyl ester	**10-OHOA-Me**	433	Phe methyl ester	Cl	H	H	OH	OH	[57]
Ethylamide Ochratoxin A	**OE-OA**	430	Phe ethyl amide	Cl	H	H	H	OH	[61]
Ochratoxin A decarboxylated	**DC-OA**	359	Phe decarboxylated	Cl	H	H	H	OH	[61]
Ochratoxin A *O*-methyl	**OM-OA**	417	Phe	Cl	H	H	H	OCH_3_	[61]
*d*-Ochratoxin A	***d*-OA**	403	*d*-Phe	Cl	H	H	H	OH	[61]
Ochratoxin α ester methyl	**M-O**α	270	OCH_3_	Cl	H	H	H	OH	[61]
Tyrosine Ochratoxin A	**OTA-Tyrosine**	419	Tyrosine	Cl	H	H	H	OH	[62]

**Table 4:** Analytical methods for determination of OTA in food, feed, and biological materials.

Method	Year	Biological Material	Limit of Detection (LOD)	References
TLC	1973	barley	12 ng/g	[68]
TLC	1973	other commodities	3–5 ng/g	[69]
spectrophotometry	1976	barley, pigs kidney, human blood (confirmation by carboxypeptidase A)	1–4 ng/g	[70]
HPLC-UVD	1979	cereals	1–5 ng/g	[71]
HPLC-FLD	1980	food and feed	5 ng/g	[72]
HPLC-FLD	1980	(confirmation by boron trifuoride methanol)		[73]
HPLC-FLD	1981	feed	1 ng/g	[74]
RIA	1975	-	20 ng/g	[75]
ELISA	1981	food, feed, biological fluids	25 pg/assay	[76]
LC-MS	1987	barley	0.5 ng/g	[77]
ion–pair HPLC	1991	human plasma	0.02 ng/mL	[78]
GC-MS	1992	food	<0.1 ng/g	[79]
HPLC-FLD	1992	corn, barley, kidney	0.2	[80]
ELISA	1993	human sera	10 pg/mL	[81]
IAC coupled with Fluorometer	1997	liquid food matrices	pg/mL	[82]
LC-ESI-MS/MS	1998	food (coffee)	20 pg/on column	[83]
LC-ESI-MS/MS	1999	pig kidney, rye flour	0.02 ng/g	[84]
HPLC-FLD Confirmation carboxypeptidase	2003	Blood, urine	0.1 ng/mL (blood); 4 ng/mL (urine)	[85]
HPLC-FLD Confirmations with carboxypeptidase + LC-MS/MS	2004	Breakfast cereal	0.05 ng/g	[86]
PFIA	2004	barley	3 ng/mL	[87]
DNA aptamer	2008	wheat	2 ng/g	[88]
LC-MS/MS	2010	urine	0.001–0.045 ng/mL	[89]
ICP-MS	2010	wine	0.003 ng/mL	[90]
LC-MS/MS	2012	urine	OTA: 0.03 ng/mL	[91]
flow electrochemical aptasensor with aptamer	2013	beer	0.05 ng/mL	[92]
UHPLC-FLR (LC-ESI-MS/MS)	2014	ginger	OTA: 0.1 ng/g; (0.005–0.2 ng/g)	[93]
LC-MS/MS	2015	dried blood spots	0.2 pg/on column	[94]
ELISA	2012	-	1.2 ng/g	[95]
Metal enhanced fluorescence	2014	Food/drinks (milk, juice)	0.5 µg/kg	[96]
Electroluminescence/Biosensor	2015	corn	0.02 pg/mL	[97]
Molecular imprinting	2015	Beer/wine	1.7 µg/L	[98]
PCR	2015	wine	19 nM	[99]

LC-ESI-MS/MS: Column liquid chromatography–electrospray ionization-tandem mass spectrometry; PFIA: Fluorescence polarization immunoassay; aptamers: Artificial short single stranded oligonucleotides, either DNA or RNA; PCR: Polymer chain reaction.

**Table 5:** OTA and alert notifications in the EU.

Date of Case	Country	Foodstuffs	OTA (ng/g)
16/01/2015	Finland	Pumpkin seeds from China	19
22/01/2015	Germany	Dried figs from Spain	124
03/03/2015	Belgium	Wheat from Canada	17
13/03/2015	Netherlands	Pumpkin seeds from China	29
13/03/2015	France	Dried figs from Spain	183
24/03/2015	France	Wheat from Canada	18
27/03/2015	Switzerland	Ground mace from Sri Lanka	42.5
12/05/2015	France	Buckwheat flour from France	40
04/06/2015	Ireland	Liquorice root from Turkey	433.5
10/06/2015	Poland	Raisins from Turkey	19.3
15/07/2015	Slovak Republic	Raisins from Chile	11.8
10/08/2015	France	Rye flour from France	12.9
12/08/2015	Finland	Pumpkin seeds from China	20000
13/08/2015	Luxembourg	Dried red chili peppers from Thailand	30.8
01/09/2015	Romania	Sultanas from Turkey	15.6
02/09/2015	Belgium	Rye malt from France	13.8
02/09/2015	Belgium	Rye malt from France	25.7
02/09/2015	Belgium	Rye malt from France	38.6
25/09/2015	Croatia	Black pepper from Vietnam	155
21/10/2015	Malta	Soft oaty bars from Switzerland	1.4
02/12/2015	Belgium	Dried figs from Turkey	14.4
08/12/2015	Latvia	Chili from China	40
11/12/2015	Cyprus	Dried sultana raisins from Greece	18.5
23/12/2015	Belgium	Dried figs from Turkey	27.8

Alert notifications are sent whenever a foodstuff presenting a serious health risk to humans is identified at the internal market and whenever the rapid action of the competent authorities is required.

**Table 6:** OTA and border rejections.

Date of Case	Country	Foodstuffs	OTA (ng/g)
22/01/2015	Poland	Raisins from Uzbekistan	21.1
26/01/2015	Netherlands	Dried figs from Turkey	24
11/02/2015	Germany	Raisins from Afghanistan	11.8
19/02/2015	Latvia	Raisins from Afghanistan	61
26/02/2015	Germany	Dried figs from Turkey	17.4
13/03/2015	Hungary	Raisins from Uzbekistan	24.3
30/06/2015	Croatia	Mixed spices from Kuwait	45
21/07/2015	United Kingdom	Red pepper powder from Ethiopia	92.5
13/08/2015	The Netherlands	Pistachios from the United States	74
31/08/2015	Germany	Berbere spice mix from Ethiopia	85.3
07/09/2015	The Netherlands	Red chili powder from India	69
28/10/2015	Poland	Red chili powder from India	32.6
16/12/2015	Germany	Red pepper spice mix from Ethiopia	69.9

Border rejections concern food and feed consignments that have been tested and rejected at the external borders of the EU.

**Table 7:** OTA and the EU Rapid Alert System for Food and Feed (RASFF) information.

Date of Case	Country	Foodstuffs	OTA (ng/g)
13/01/2015	Germany	Dried figs from Turkey	69.9
16/01/2015	Germany	Dried figs from Turkey	45
16/02/2015	Germany	Sun dried figs from Turkey	86
17/02/2015	Germany	Dried figs from Turkey	32
02/06/2015	Germany	Spice mix and paprika from Ethiopia	139
24/07/2015	Denmark	Organic raisins from Australia	28
23/12/2015	Germany	Dried figs from Turkey	10.8

Food that is only present in the notifying EU Member State is worth noting.

**Table 8:** Nephrotoxicity of OTA.

Year	Nephrotoxicity Testing	References
1972	Balkan endemic nephropathy (BEN) has been suggested to be the result of fungal poisoning. The role of OTA in mycotoxicosis—BEN in humans and porcine nephropathy.	[156]
1972	In view of the similarities between BEN and OTA induced porcine nephropathy, it has been suggested that OTA may be involved in the etiology of BEN.	[157]
1978	OTA is potentially nephrotoxic in all species tested with the exception of adult ruminants.	[158]
1987	Findings of higher OTA levels in the serum of patients suffering from BEN, which is a subtype of tubulointerstitial nephritis, led to hypotheses about the association between the nephrotoxicity of OTA and the BEN and also the incidence of renal system tumors in the population of these Balkan regions.	[159]
1991	Nephropathy is primarily related to the mobilization of intracellular calcium.	[160]
1992	In terms of human pathologies, OTA is suspected to be the main etiological agent responsible for BEN and associated urinary tract tumors (UTT) in humans.	[161]
1993	Experimental studies on the nephrotoxicity of OTA both in vitro and in vivo have shown that OTA disturbs the intracellular metabolic processes (with subsequent apoptosis of the renal cells), renal hemodynamics, and—significantly and perhaps preponderantly—the functions of the proximal tubules (even after subchronic exposition). OTA causes the decrease of glomerular filtration and tubular resorption and affects all parts of the nephron and kidneys in toto.	[162,163,164,165,166,167,168]
1993	A case of acute nephrotoxicity in humans.	[169,170]
1999	OTA induces apoptosis in cultured human proximal tubule cells.	[171]
2002–2005	The kidney is the main target of OTA toxicity in all animal species tested.	[14,172]
2002–2005	OTA has been also implicated in the etiology of BEN, a chronic degenerative kidney disease, in kidney tumors in humans in certain regions of the Balkan Peninsula, and in chronic interstitial nephropathy (CIN) in Tunisia and other North African countries.	[14,148,150]
2005	Exposure to low OTA doses is responsible for nephrotoxicity; at nanomolar concentrations, OTA leads to specific changes of function and phenotype in renal cells.	[173]
2007–2010	Very low OTA concentrations administered for a prolonged time (up to 14 days) influence the cellular fate (cellular hypertrophy) in human proximal tubule; furthermore, they act not only in the target organ, e.g., in the kidney, but also in as yet unsuspected cells, such as fibroblasts; the same damage will likely occur in chronic exposure.	[174,175]
2013	Nephrotoxicity is a consequence of acute, sub-acute, and also chronic exposure to OTA.	[9]
2014	OTA inhibits the nuclear factor, erythroid 2-like 2 (Nrf2) oxidative stress response pathway. Nrf2 overexpression confers a survival advantage and is often associated with cancer cell survival.	[176]
2015	Dietary exposure to OTA represents a serious health issue including, e.g., human endemic nephropathies.	[50]

**Table 9:** OTA carcinogenicity and genotoxicity.

Year	Nephrotoxicity testing	References
1978	OTA induces renal and hepatic tumors in mice.	[177]
1984	OTA is carcinogenic for mice.	[178]
1984	CIT increases OTA carcinogenicity.	[179]
1987	OTA carcinogenicity to humans: OTA classified in Group 3 (not classifiable as to its carcinogenicity to humans).	[180]
1989	Male rats are more susceptible to renal tumors than female rats (NTP study).	[181]
1989	The genotoxicity of ochratoxin A is reviewed.	[35,182]
1991	OTA-DNA adducts: For the first time, OTA-DNA adducts are found in the kidney, liver, and spleen of mice.	[183]
1993	OTA is re-classified as a possibly carcinogenic to humans based on a great amount of evidence of carcinogenity in several animal studies of 2B in 1993.	[11]
1993	OTA-DNA adducts: Other studies take place in mice and rat tissues after acute and subchronic exposure, and in urinary tract tumors (UTT) of Bulgarian subjects.	[184,185,186]
1993-2009	OTA-DNA adducts are also detected in tissues of humans presumably exposed to OTA in several countries (Bulgaria, Serbia, Croatia, Germany, Belgium, France, Tunisia).	[16,17,185,187,188,189,190]
1998-2002	DNA adduction following chronic exposure (carcinogenic study) of rats to OTA first described; sex differences and dual mechanism—oxidative pathways and DNA adduction—are observed	[12,13,191]
1998	OTA-DNA adducts are observed in mother and progeny of mice fed OTA nine months after birth male mice develop cancer.	[192]
2000–2001	In vitro formation of dG-OTA adduct.	[193,194]
2001–2002	Other studies with radiolabeled OTA were unable to detect any DNA binding of OTA, but explanation of this discrepancy is given in depth by Pfohl-Leszkowicz and Castegnaro in 2005 [ 195]	[60,196]
2003	OTA-DNA adduct in pigs subchronically exposed to low doses of OTA. Relation with biotransformation.	[197]
2002–2010	OTA may be involved in testicular cancer.	[175,198,199,200,201]
2003–2008	CIT increases genotoxicity of OTA and modifies the metabolism of rats exposed to low doses for three weeks.	[202,203]
2004	Evidence for covalent DNA adduction by OTA following chronic exposure to OTA in rats (and subacute exposure in pigs).	[190]
2004	Another research group, using the highly sensitive accelerator of the mass spectrometry technique, does not detect DNA adducts after the administration of ^14^*C*-labeled OTA to rats.	[204]
2004	In 2004, a review of the NTP experimental rat tumor data for OTA also places OTA in the category of “chemicals inducing renal tumors through direct interaction of the parent compound or metabolite with renal DNA” based on histopathological evidence.	[205]
2004–2010	The long-term OTA studies confirm the incidence of tumors in rats; in male rats, these tumors are related to OTA dose	[205,206,207]
2004–2012	OTA is a direct genotoxic forming covalent DNA adducts in the kidney OTA can indeed react with DNA via a phenolic radical resulting in C8-deoxyguanosine adduct (synthetized and chemical identified by mass spectrum).	[175,190,201,207,208,209]
2006	Confirmation of OTA genotoxicity via measurement of comet in rat kidneys.	[210]
2007	Chronic exposure to low OTA doses can be much more damaging than acute exposure to a high dose.	[16]
2007	DNA diploidy in rat tumors is associated to genetic damage.	[211]
2007	OTA induces an increase of mutation at two loci—hypoxantine-guanine phophoribosyl transferase (HPRT) and thymidine kinase (TK).	[212]
2008	DNA adduct cannot be confirmed, but the explanation is given by Pfohl-Leszkowicz et al. (2009) [64]	[213]
2008	Correlation between biotransformation of OTA and direct covalent binding on DNA.	[214]
2009	It is found that the kidney DNA adduct pattern of BEN patients is similar to the kidney DNA adduct pattern of pigs living in the same farm and pigs co-exposed to OTA, fumonisins, and citrinin.	[17]
2009	A different proposal of mechanism for OTA-mediated renal carcinogenesis and threshold model for its risk assessment.	[215]
2009–2010	Identification by LC-MS/MS of these DNA adduct in rat tissues.	[64,201]
2010	OTA is carcinogenic for poultry.	[216]
2011	Induction of mutation only in medulla of rat kidney exposed to carcinogenic dose.	[217]
2012	Relation structure activity studies clearly indicate that OTHQ (ochratoxin hydroxyquinone) is responsible of direct genotoxicity, whereas some others are cytotoxic.	[65,209]
2012	OTA is activated to a species that is a directly genotoxic mutagen. OTHQ in presence of cysteine is also mutagenic.	[218]
	A new approach to cancer represents miRNA.	[219,220]
2013	The induction of miR-132 and miR-200c by OTA elevates reactive oxygen species (ROS) levels and profibrotic (profibrotic transforming growth factors β, TGFβ) expression.	[221]
2014	OTA has the potential to initiate or support the development of fibrotic kidney diseases by involving post-transcriptional regulation mechanisms comprising miR-29b. OTA reduces the impact of miR-29b and thus enhances collagen protein expression.	[222]
2014	A low dose of OTA induces micronuclei, and OTA delays the DNA repair kinetics.	[223]
2014	OTA increases proliferating cell nuclear antigen after 13 weeks in kidney and kidney damages. Limited oxidative stress.	[224]
2015	Dietary exposure to OTA represents a serious health issue, including urinary tract tumors in humans.	[50]

**Table 10:** An overview of chronologically published data on OTA in blood samples from healthy persons.

Country	Collecting Period	n+ (%)	OTA min–max (μg/L)	OTA Mean (μg/L)	Reference
*Europe*					
Former Yugoslavia	1980	7.8	max. 8.0	5.4	[229,241]
Germany	1977–1985	56.5	0.1–14.4	0.6	[242]
Bulgaria	1984,1986, 1989–1990	10	-	12.0	[243,244]
Poland	1983–1985	7.2	1–40	0.28	[245]
Former Yugoslavia	1981–1989	0-3.7	max. 50.0	-	[246]
Germany	1988	68	0.1–8.4	0.75	[247]
Sweden	1989	12.8	0.3–7.0	0.20	[78]
Czechoslovakia	1990	21	0.5–12.0	0.37	[248]
Denmark	1990	54.2	0.1–13.2	1.8	[241]
France	-	-	0.1–6.0 (rural); 0.1–1.3 (urban)	-	[249]
Czechoslovakia	1990–1991	40	0.5–19.4	0.63	[250]
France	1991–1992	18.1	0.1-161	0.4	[251,252]
Italy	1992	100	0.1–2.0	0.53	[253]
Switzerland	1992–1993	100	0.06–6.02	ca. 0.4	[105]
Hungary	1995	51	0.2–12.9	-	[254]
Italy	1994–1996	97	0.1–57.2	0.56	[255]
Hungary	1995	82	0.2–10.0	-	[256]
Czech Republic	1994–2002	94.2	0.1–13.7	0.24	[257,258,259,260]
Spain	1996–1998	53.3	0.5–4.0	0.71	[261]
Spain	1996–1997	72	0.21–6.96	0.63	[262]
Hungary	1997	77	0.1–1.4	-	[263]
Croatia	1997–1998	59.4	max. 15.9	0.30	[264,265,266]
Sweden	1997	100	0.01–0.48	0.21	[145,267]
Norway	1998	100	0.05–0.42	0.18	[145,267]
Germany	1999	98.1	0.06–2.03	0.27	[268]
UK	2000	100	0.4–3.11	1.09	[145,269]
Norway	-	-	0.02–5.53	0.40	[270]
Bulgaria	-	100	max. 8.4	1.59	[85]
Portugal	2001–2002	100	0.14–2.49	-	[271]
Poland	2005	100	0.1–0.4	0.37	[272]
Germany	2005–2006	100	0.05–0.75	0.75	[18]
Czech Republic	2005	83.7	0.1–2.3	0.21	[273]
Spain	2008	100	0.15–5.71	1.09	[274]
Spain	2008	98.6	0.11–8.68	0.86	[275]
Germany	2008	100	0.19–0.29	0.25	[276]
Spain	-	100	0.06–10.92	0.8	[277]
Italy	-	99.1	0.03–2.92	0.23	[278]
Czech Republic	2012	96	0.1–0.35	0.15	[279]
Czech Republic	2012	-	0.37–1.13	0.17	[280]
*Africa*					
Algeria	-	66.9	max. 9.0	2.8	[281]
Tunisia	-	62	max. 3.2	1.22	[149]
	-	66	max. 2.3	1.1	[282]
Egypt	-	2.9	max. 0.91	0.08	[151]
Sierra Leone	1996	33	max. 18.2	-	[283]
Morocco	2000	60	0.08–6.59	0.2	[284]
	1991–2000	62-82	0.1–5.5	2.0	[285]
	1996, 1998	100	0.1–8.06	0.53	[150]
	-	71	max. 7.5	2.6	[286]
Ivory Coast	2001, 2004	34.9	max. 11.62	0.58	[287]
Tunisia	-	28	0.12–3.4	0.49	[288]
Tunisia	-	52.3	0.11–6.1	0.77	[289]
Tunisia	2007–2009	49	1.7–8.5	3.3	[290]
Tunisia	-	34	0.12–1.5	0.22	[291]
*Asia*					
Japan	1992-1996	85	max. 0.28	0.07	[292]
Lebanon	2001-2002	33	max. 1.24	0.31	[293]
Pakistan	-	97	max. 1.24	0.31	[294]
Turkey	-	-	max. 1.43	0.44	[295]
Turkey	2008–winter	76.7	0.03–0.89	0.14	
	2007–summer	97.5	0.03–1.50	0.31	[296]
Bangladesh	-	100	0.2–6.63	0.85	[240]
Turkey	–summer	100	0.03–1.55	0.31	
	–winter	83.3	0.05–1.12	0.5	[297]
*The Americas*					
Canada	1991	38.3	max. 9.0	1.29	[298]
Canada	1994*	100	max. 2.37	0.88	[299]
Chile	2004	54	0.4–2.75	0.44	
(2 regions)		91	0.4–2.12	0.77	[300]
Costa Rica	-	95	max. 1.91	0.62	[301]
Argentina	2004–2005	63.8	0.19–47.6	0.15	
(2 regions)			0.19–74.8	0.43	[302]

Abbreviations: n+ (%): percentage of positive samples; *study included persons working at grain storage facilities; rural, urban (population).

**Table 11:** The results of OTA in human morning urine from different populations.

Country	n	n+ %	Mean (ng/L)	Reference
Croatia	35	94	239.0	[311]
Hungary	88	61	13.0	[312]
Portugal	60	70	27.0	[313]
Portugal	30	43	19.0	[314]
Portugal	43	72.1	26.0	[315]
Croatia	45	43	17.0	[316]
Croatia	45	18	7.0	[316]
Portugal	155	92	18.0	[317]
Turkey	233	90	14.3 *	[318]
Germany	13	100	70.0	[276]
South Korea	12	100	31.0	[89]
Spain	72	12.5	237.0	[319]
Spain	27	no stated	-	[320]
Italy	10	100	-	[321]
Sri Lanka	31	93.5	20.0 **	[322]
Portugal	95	87.4	22.0 (winter)	[323]
Portugal	95	81.1	16.0 (summer)	[323]
Croatia	40	78.0	90.0 (before enzyme treatment)	[324]
Croatia	40	58.0	130.0 after enzyme treatment)	[324]
Cameroon	175	63	280.0	[308]
Cameroon	145: HIV positive	17	80.0	[325]
	30: HIV: sero-negative	10	60.0	
South Africa	53	98	41.0	[326]
Cameroon	220	32	200.0	[309]
Italy	52	100	144.0	[327]
Chile	39		30–433 *** 30–124 ****	[239]
Portugal	472	86.4	19.0 *****	[328]
Germany	30	15	40.0	[329]
Haiti	47	33	109.0	[329]
Bangladesh	72	76	203.0	[329]

Abbreviations: n: numbers of samples; n+ %: percentage of positive samples; * ng/g creatinine; ** GM: geometric mean; *** range in newborns consuming colostrums; **** range of samples collected between 4 and 6 months of infants’ life; ***** mean in ng/kg.

**Table 12:** Data on OTA in human milk worldwide.

Country	n	n+ (%)	Range Positive Samples (ng/L)	References
*European countries*				
Germany	36	11	17–30	[330]
Italy	50	18	1,200–6,600	[332]
Sweden	40	58	10–40	[331]
Hungary	92	41	200–7,200	[255]
Switzerland	40	10	5–14	[105]
Italy	111	20	100–12,000	[334]
Italy	4	75	8-540	[335]
Norway	115	33	10–130	[336]
Norway	80	21	10–182	[333]
Italy	231	86	10–57	[337]
Poland	13	38	6–17	[338]
Italy	82	74	5–405	[339]
Slovakia	76	30	2–60	[340]
Italy	57	78.9	1–75	[341]
Germany	90	60	10–100	[342]
*Africa*				
Sierra Leone	113	35	200–337,000	[343]
Egypt	120	36	5,000–45,000	[344]
Egypt	50	72	1,890 ± 980 *	[345]
*Australia*	100	2	3,000–3,600	[346]
*Asia*				
Turkey	75	100	620–13,111	[347]
Iran	136	2.7	90–140	[348]
Iran	87	84	1.6–60	[349]
*The Americas*				
Brazil	50	4	10–20	[350]
Chile	11	100	44–184	[351]
Brazil	224	0		[352]
Chile	50	80	10–186	[239]
Brazil	100	66	0.3–21	[353]

*: no ranges were provided.

**Table 13:** The first maximum levels of OTA in foodstuffs under Regulation 466/2001 as amended by Regulation 472/2002.

Foodstuffs	Maximum levels (ng/g)
Cereals (including rice and buckwheat) and derived cereal products	5
Raw cereal grains (including raw rice and buckwheat)	5
All products derived from cereals (including processed cereal products and cereal grains intended for direct human consumption)	3
Dried vine fruit (currants, raisins and sultanas)	10
Green and roasted coffee and coffee products, wine, beer, grape juice, cocoa and cocoa products, and spices	-

**Table 14:** Maximum levels of OTA in foodstuffs under Regulation 1881/2006 as in force.

Code	Foodstuffs	Maximum Levels (ng/g)
2.2.1	Unprocessed cereals	5.0
2.2.2.	All products derived from unprocessed cereals, including processed cereal products and cereals intended for direct human consumption with the exception of foodstuffs listed in 2.2.9, 2.2.10, and 2.2.13	3.0
2.2.3	Dried vine fruit (currants, raisins, and sultanas)	10.0
2.2.4	Roasted coffee beans and ground roasted coffee, excluding soluble coffee	5.0
2.2.5	Soluble coffee (instant coffee)	10.0
2.2.6	Wine (including sparkling wine, excluding liqueur wine and wine with an alcoholic strength of not less than 15 vol %) and fruit wine	2.0
2.2.7	Aromatized wine, aromatized wine-based drinks, and aromatized wine-product cocktails	2.0
2.2.8	Grape juice, concentrated grape juice as reconstituted, grape nectar, grape must and concentrated grape must as reconstituted, intended for direct human consumption	2.0
2.2.9	Processed cereal-based foods and baby foods for infants and young children	0.50
2.2.10	Dietary foods for special medical purposes intended specifically for infants	0.50
2.2.11.	Spices, including dried spices	
	*Piper* spp. (fruits thereof, including white and black pepper), *Myristica fragrans* (nutmeg), *Zingiber officinale* (ginger), *Curcuma longa* (turmeric)	15
	*Capsicum* spp. (dried fruits thereof, whole or ground, including chilies, chili powder, cayenne, and paprika)	20
	Mixtures of spices containing one of the abovementioned spices	15
2.2.12.	Liquorice (*Glycyrrhiza glabra, Glycyrrhiza inflate* and other species)	
2.2.12.1.	Liquorice root, ingredient for herbal infusion	20
2.2.12.2.	Liquorice extract for use in food in particular beverages and confectionary	80
2.2.13.	Wheat gluten not sold directly to the consumer	8.0

**Table 15:** Guidance values for OTA under Commission Recommendation 2006/576/EC as in force.

Feed	Guidance Value in mg/kg Relative to Feedstuffs with a Moisture Content of 12%
Feed materials *—Cereals and cereal products **	0.25
Complementary and complete feedstuffs	
—Complementary and complete feedstuffs for pigs	0.05
—Complementary and complete feedstuffs for poultry	0.1

* Particular attention must be paid to cereals and cereals products fed directly to the animals that their use in a daily ration should not lead to the animal being exposed to a higher level of these mycotoxins than the corresponding levels of exposure where only the complete feedstuffs are used in a daily ration. ** The term “Cereals and cereal products” includes not only the feed materials listed under Heading 1, “Cereal grains, their products and by-products,” of the non-exclusive list of main feed materials referred to in Part B of the Annex to Council Directive 96/25/EC of 29 April 1996 on the circulation and use of feed materials (OJ L 125, 23.5.1996, p. 35), but also other feed materials derived from cereals in particular cereal forages and roughages.

## Abbreviations

10-OHOA: 10-hydroxy ochratoxin A
10-OHOA-Me: 10-hydroxy ochratoxin A methyl ester
2′-DC-OTA: 2′-ochratoxin A decarboxylated
2′R-OTA: 2′R-ochratoxin A
4R-OHOA: 4R-hydroxy ochratoxin A
4R-OHOA-Me: 4R-hydroxy ochratoxin A methyl ester
4S-OHOA: 4S-hydroxy ochratoxin A
Acyl-hexose-OTA: conjugate ochratoxin A–acyl hexose
Acyl-pentose OTA: conjugate ochratoxin A–acyl pentose
BEN: Balkan endemic nephropathy
CAC: Codex Alimentarius Commission
CAS: Chemical Abstracts Services
CE-LIF: capillary electrophoresis with laser-induced fluorescence detection
CIN: chronic interstitial nephropathy
CIT: citrinin
DC-OA: ochratoxin A decarboxylated
DC-OTHQ: OTHQ decarboxylated
DNA aptamer: Artificial short single stranded oligonucleotides
DNA: Deoxyribonucleic acid
d-OA: d-ochratoxin A
EU: European Union
FB: fumonisin
FDA: Food and Drug Administration
FFDCA: Federal Food, Drug, and Cosmetic Act
GC-MS: gas chromatography–mass spectrometry
HPLC-FLD: high-performance liquid chromatography with fluorescence detection
HPLC-UVD: high-performance liquid chromatography with ultraviolet detection
IAC: immunoaffinity columns
TGFβ: profibrotic transforming growth factors β
ROS: reactive oxygen species
IARC: The International Agency for Research on Cancer
ICP-MS: inductively coupled plasma mass spectrometry
IgE: immunoglobulin E
IgG: immunoglobulin G
IgM: immunoglobulin M
IPCS: International Programme on Chemical Safety
IUPAC: International Union of Pure and Applied Chemistry
JECFA: The Joint FAO/WHO Expert Committee on Food Additives
LC-ESI-MS/MS: column liquid chromatography electrospray ionization tandem mass spectrometry
LC-MS: liquid chromatography–mass spectrometry
LC-MS/MS: liquid chromatography-tandem mass spectrometry
LOD: limit of detection
LOQ: limit of quantification
MEKC: micellar electrokinetic capillary chromatography
MIPs: molecular imprinted polymers
M-Oα: Ochratoxin α ester methyl
OE-OA: ethylamide ochratoxin A
OM-OA: ochratoxin A O-methyl
OP-OTα: ochratoxin α open lactone
OP-OA: ochratoxin A open lactone
OP-OB: ochratoxin B open lactone
OP-OTα: ochratoxin α open lactone
OTα: ochratoxin α
OTβ: ochratoxin β
OTA: ochratoxin A
OTA-Me: ochratoxin A methyl ester
OTA-Tyrosine: tyrosine ochratoxin A
OTB: ochratoxin B
OTB-Et: ochratoxin B ethyl ester
OTB-Me: ochratoxin B methyl ester
OTC: ochratoxin C
OTHQ: ochratoxin A hydroquinone
OTQ: ochratoxin A quinone
OTQ-Glutathion: conjugate ochratoxin A quinone–glutathion
PCR: polymerase chain reaction
PTWI: provisional tolerable weekly intake
PFIA: fluorescence polarization immunoassay
RASFF: Rapid Alert System for Food and Feed
RIA: radioimmunoassay
RNA: ribonucleic acid
SPE: solid-phase extractions
TDI: tolerable daily intake
TLC: solid thin layer chromatography
TTIP: The Transatlantic Trade and Investment Partnership
TWI: tolerable weekly intake
UTT: urinary tract tumors
WHO: World Health Organization
WTO: World Trade Organization
**EDI**: **exposure daily intake**
